# Supplementary material for: Utilizing Nanopore direct RNA sequencing of blood from patients with sepsis for discovery of co- and post-transcriptional disease biomarkers
Source: BMC Infect Dis. 2025 May 13;25:692. doi: 10.1186/s12879-025-11078-z (PMC12070577; doi:10.1186/s12879-025-11078-z)
Supplement: Supplementary file 1 — Additional File 1: Figures. Supporting supplementary figures 1-9. [file 12879_2025_11078_MOESM1_ESM.pdf]

SUPPLEMENTARY FIGURES

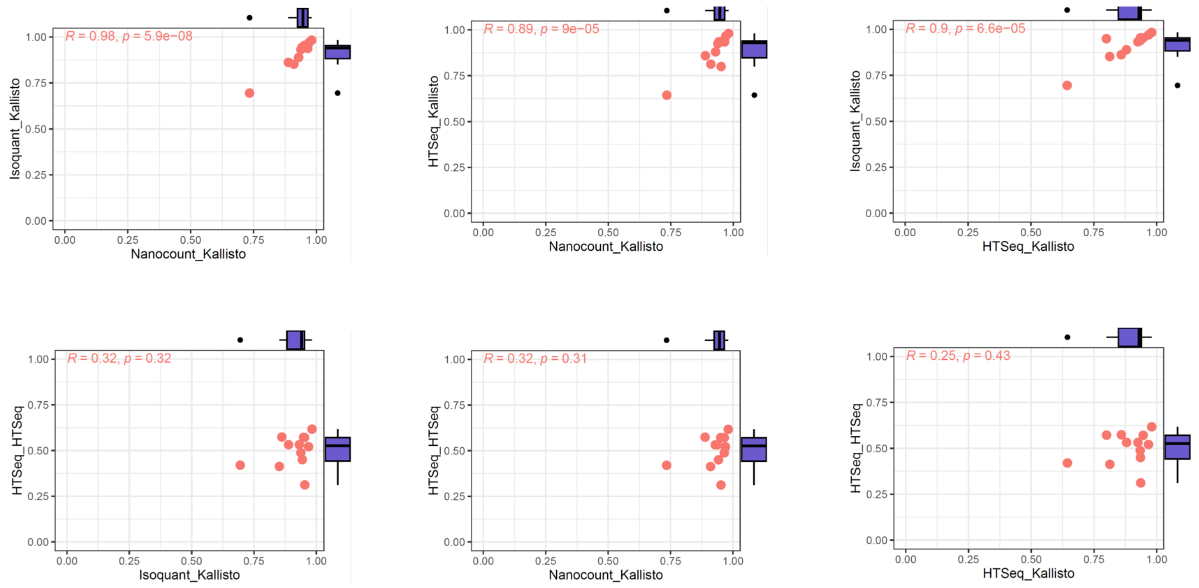

Supplementary Figure 1. The correlation plots for each pair of software comparisons between Nanopore direct RNA-seq and Illumina cDNA-seq.

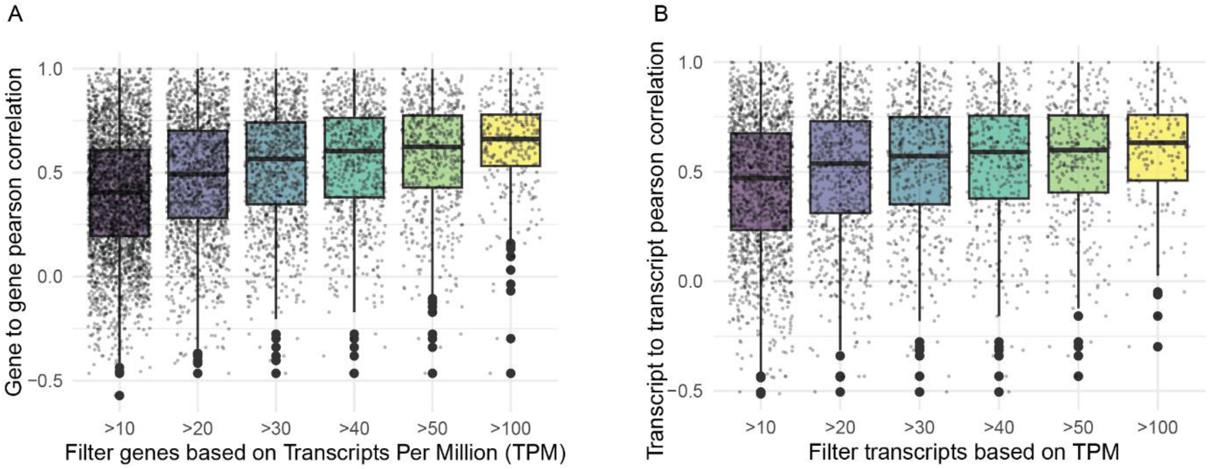

Supplementary Figure 2. Comparison with Direct RNA-seq and Illumina cDNA-seq of matching 12 samples using different pipelines. *NanoCount* for Nanopore and *Kallisto* for Illumina sequencing data were implemented, where Pearson correlations with **A)** gene-to-gene and **B)** transcript-to-transcript are shown. The X-axis indicates filter thresholds of the genes based on the level of TPM, and the Y-axis indicates the Pearson correlations.

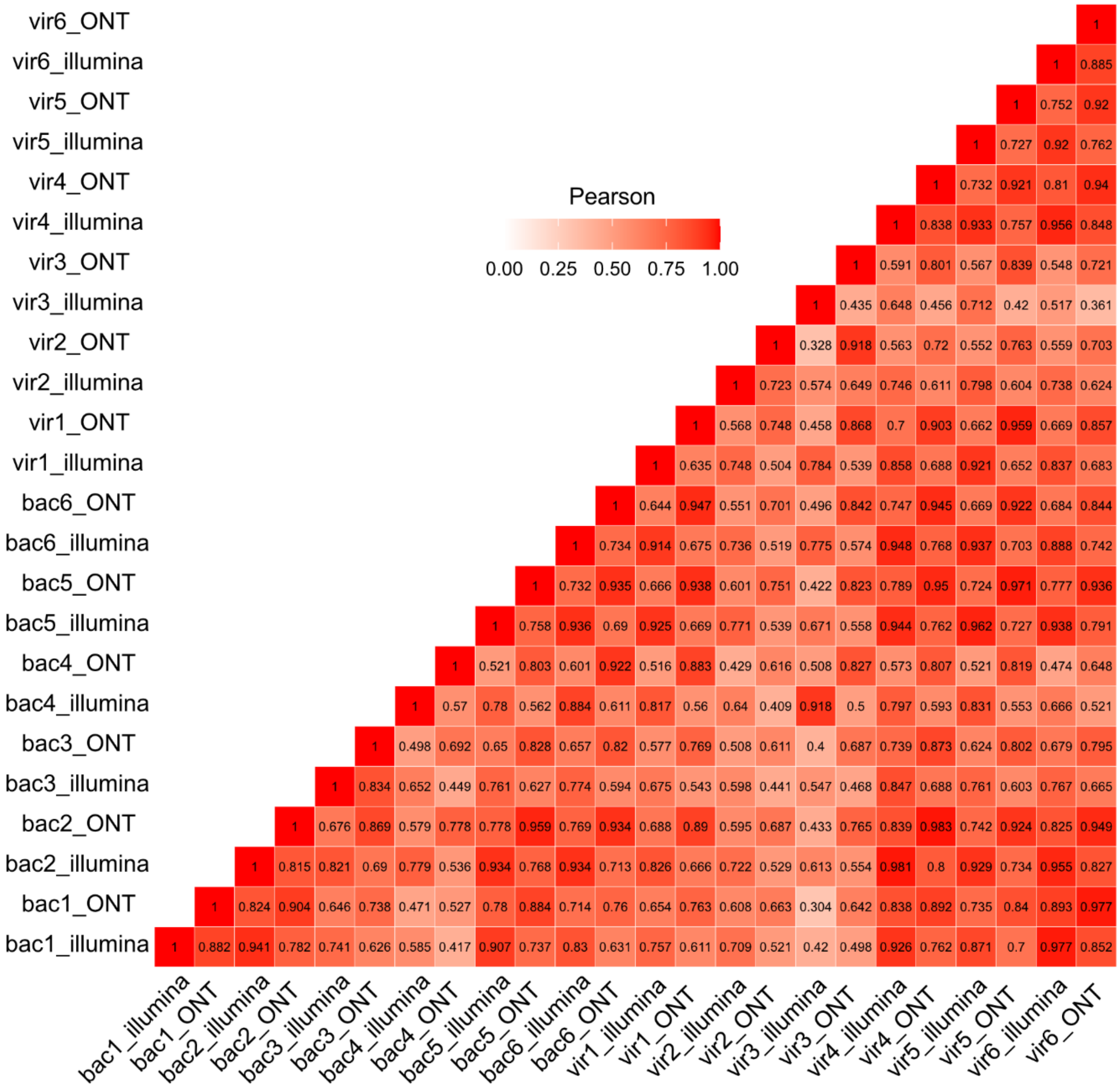

Supplementary Figure 3. The heatmap of Pearson correlations on coding genes at the transcript level across all 12 samples using *NanoCount* for Nanopore and *Kallisto* for Illumina cDNA-seq.

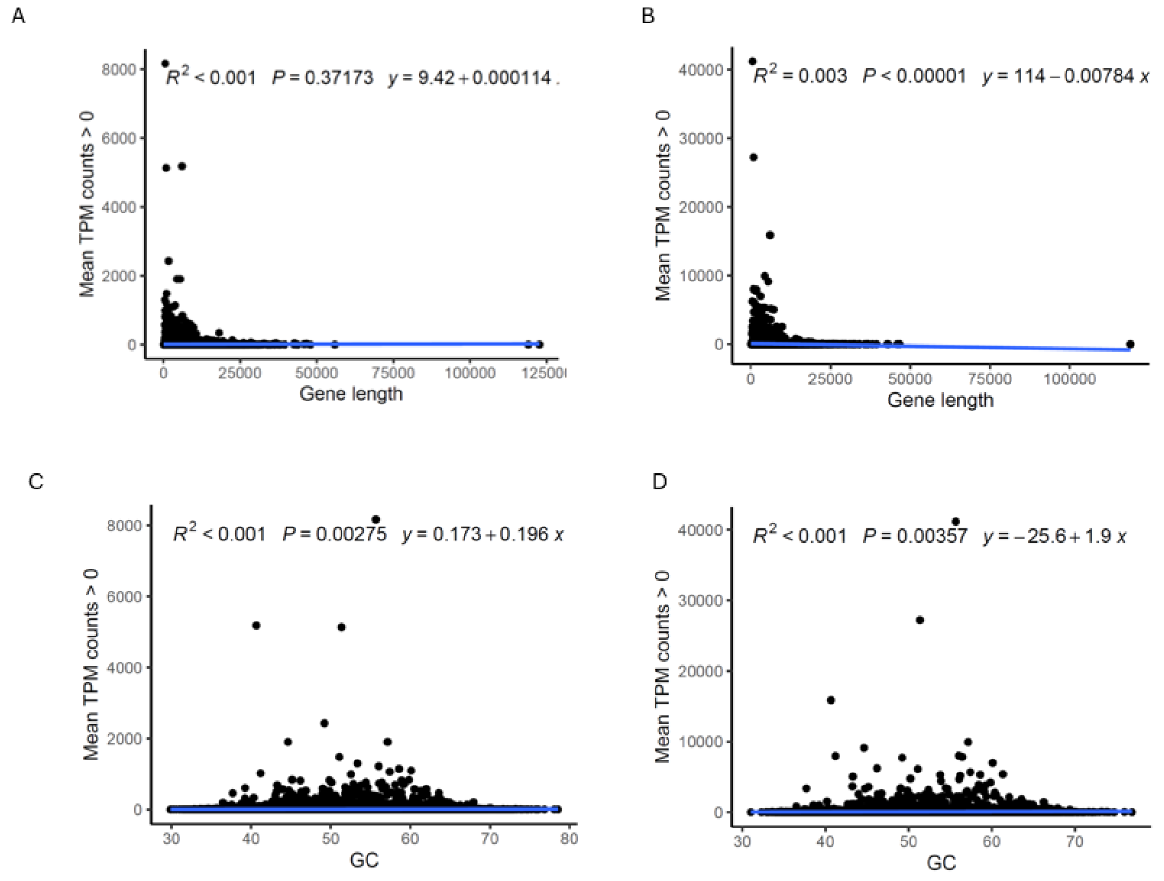

Supplementary Figure 4. The linear regression between the length of genes and GC percentages with expression level from different platforms. (A-B) The length of genes with TPM counts from **A)** Illumina cDNA-seq from *Kallisto* and **B)** Nanopore RNA-seq from *NanoCount*. **(C-D)** The GC percentages with TPM counts from **C)** Illumina cDNA-seq from *Kallisto* and **D)** Nanopore RNA-seq from *NanoCount*.

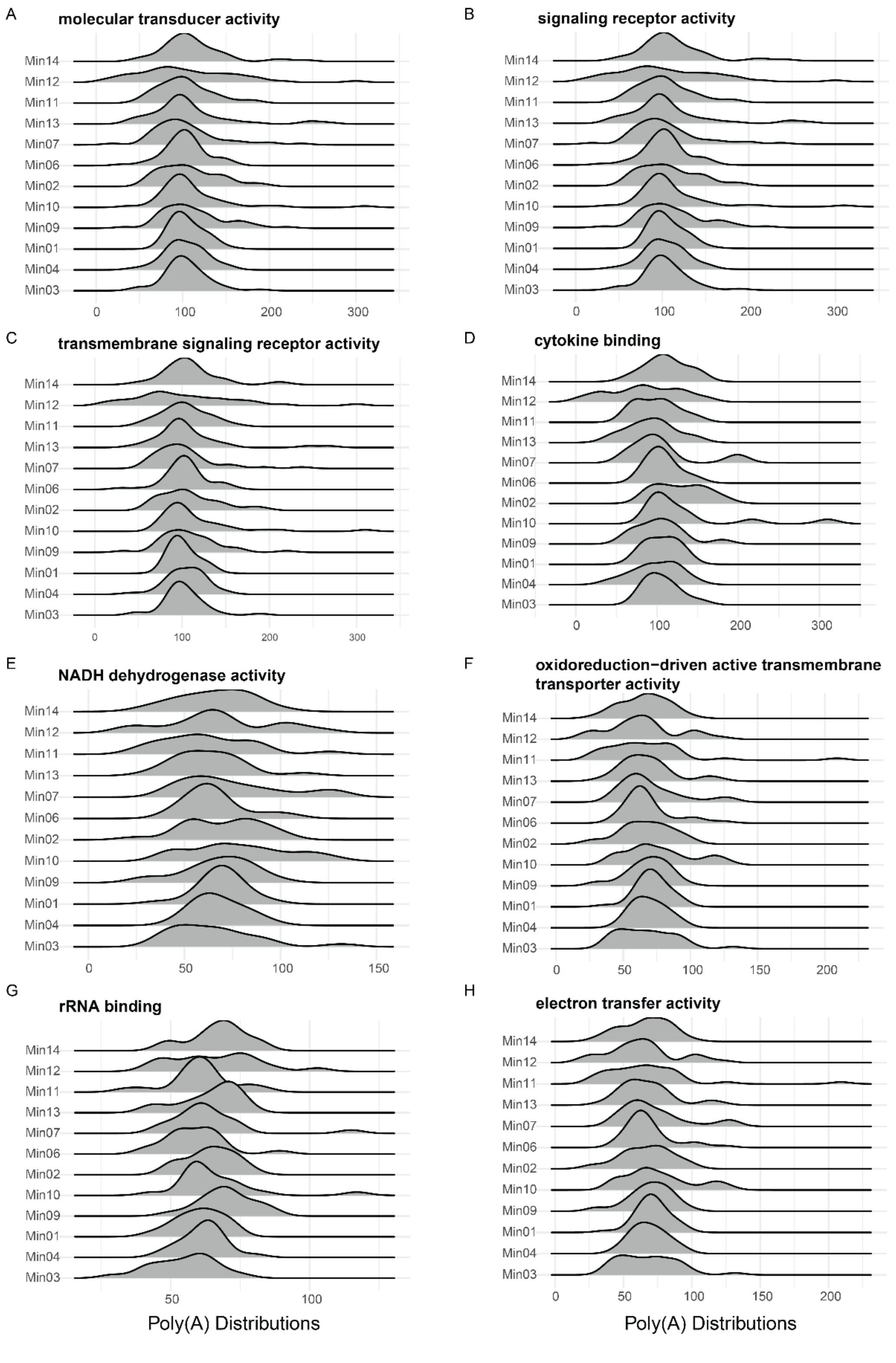

Supplementary Figure 5. The poly(A) distributions for some top molecular functional pathways across 12 different samples. (A-D) The top molecular functional pathways enriched with relatively longer poly(A) tails. **(E-H)** The top molecular functional pathways enriched with relatively shorter poly(A) tails. Each plot is labeled with the name of the corresponding molecular functional pathway. The X-axis indicates the poly(A) length distributions in nt and the Y-axis shows the individual data of the 12 samples.

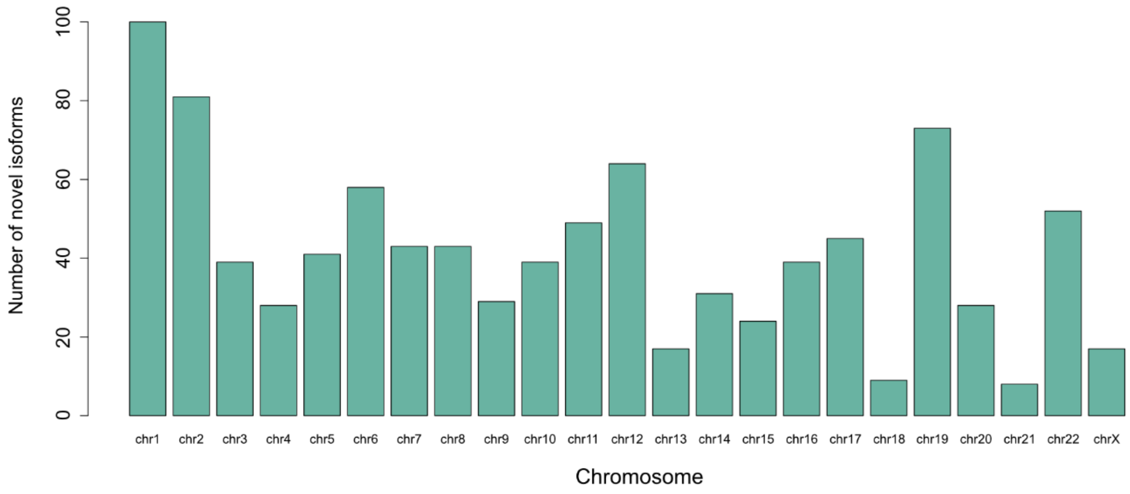

Supplementary Figure 6. Number of novel isoforms identified by *IsoQuant* and *SQANTI3* per chromosome.

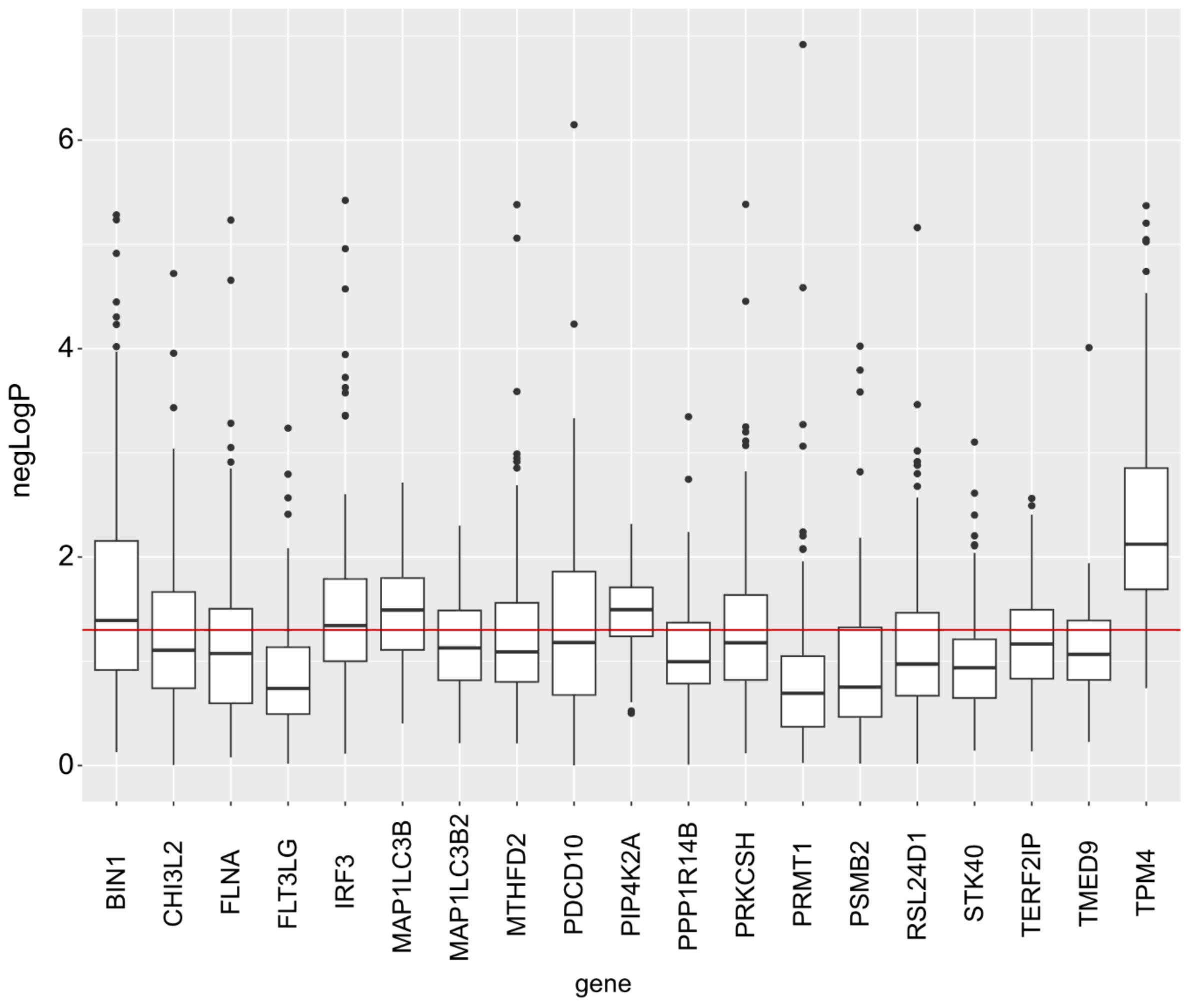

Supplementary Figure 7. Bootstrapping results for 19 DPGs from linear mixed-effects testing. The X-axis shows the DPGs and Y-axis shows the –log adjusted P-values, with the red horizontal line representing the threshold of adjusted P-value of 0.05. The results show that *TPM4* and *PIP4K2A* have robust DP between bacterial and viral infection.

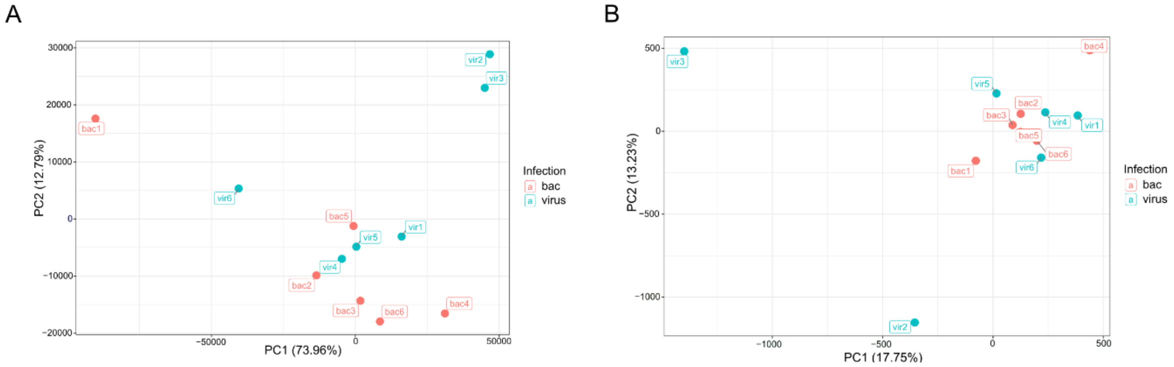

Supplementary Figure 8. (A-B) PCA plot of all 12 samples based on A) *NanoCount* expression levels and B) poly(A) tail-lengths.

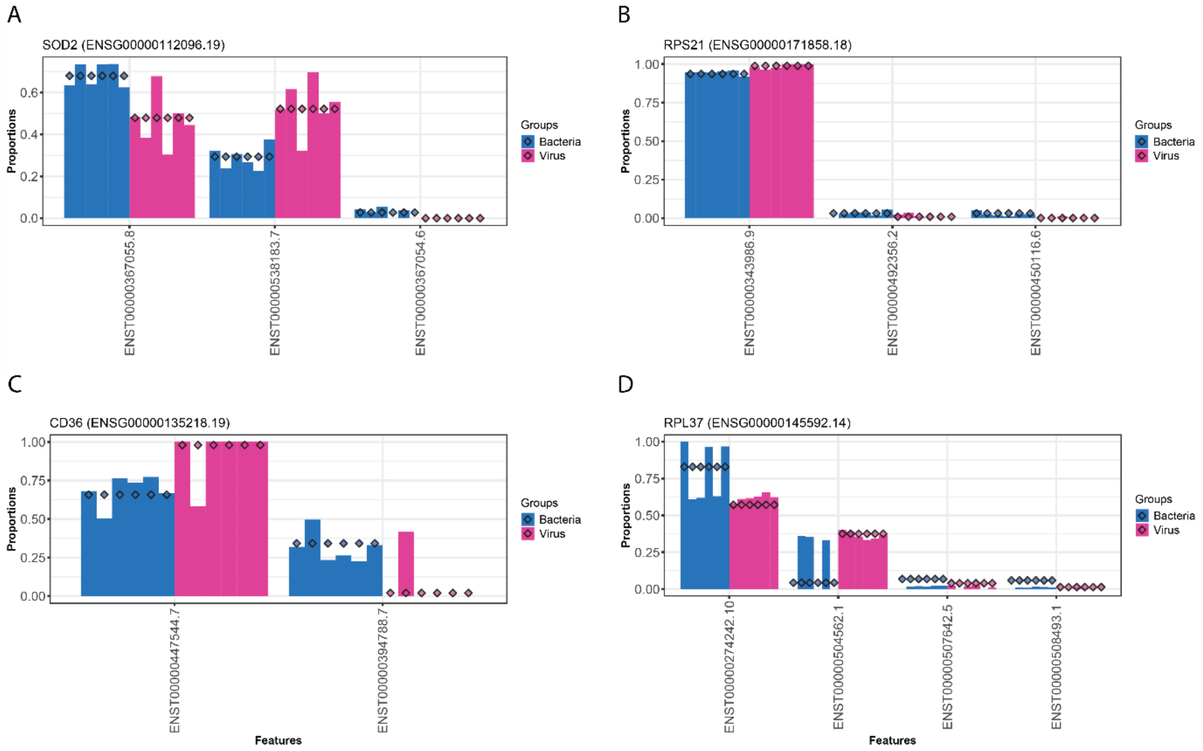

Supplementary Figure 9. Differential transcript usage occurs between bacterial and viral samples. (A-D) Differential estimated proportions of transcripts of genes for **A)** *SOD2 (ENSG00000112096.19)*, **B)** *RPS21(ENSG00000171858.18)*, **C)** *CD36 (ENSG00000135218.19)*, and **D)** *RPL37 (ENSG00000145592.14)*, with adjusted P-values < 0.05.
